# Uncoupling between Inflammatory and Fibrotic Responses to Silica: Evidence from MyD88 Knockout Mice

**DOI:** 10.1371/journal.pone.0099383

**Published:** 2014-07-22

**Authors:** Sandra Lo Re, Yousof Yakoub, Raynal Devosse, Francine Uwambayinema, Isabelle Couillin, Bernard Ryffel, Etienne Marbaix, Dominique Lison, François Huaux

**Affiliations:** 1 Louvain centre for Toxicology and Applied Pharmacology (LTAP), Institut de Recherche Expérimentale et Clinique (IREC), Université catholique de Louvain, Brussels, Belgium; 2 University of Orleans, CNRS, UMR7355, INEM, Transgenose Institute, Orleans, France; 3 de Duve Institute, Université catholique de Louvain, Brussels, Belgium; University of Rochester Medical Center, United States of America

## Abstract

The exact implication of innate immunity in granuloma formation and irreversible lung fibrosis remains to be determined. In this study, we examined the lung inflammatory and fibrotic responses to silica in MyD88-knockout (KO) mice. In comparison to wild-type (WT) mice, we found that MyD88-KO animals developed attenuated lung inflammation, neutrophil accumulation and IL-1β release in response to silica. Granuloma formation was also less pronounced in MyD88-KO mice after silica. This limited inflammatory response was not accompanied by a concomitant attenuation of lung collagen accumulation after silica. Histological analyses revealed that while pulmonary fibrosis was localized in granulomas in WT animals, it was diffusely distributed throughout the parenchyma in MyD88-KO mice. Robust collagen accumulation was also observed in mice KO for several other components of innate immunity (IL-1R, IL-1, ASC, NALP3, IL-18R, IL-33R, TRIF, and TLR2-3-4,). We additionally show that pulmonary fibrosis in MyD88-KO mice was associated with the accumulation of pro-fibrotic regulatory T lymphocytes (T regs) and pro-fibrotic cytokine expression (TGF-β, IL-10 and PDGF-B), not with T helper (Th) 17 cell influx. Our findings indicate that the activation of MyD88-related innate immunity is central in the establishment of particle-induced lung inflammatory and granuloma responses. The development of lung fibrosis appears uncoupled from inflammation and may be orchestrated by a T reg-associated pathway.

## Introduction

Silicosis is a lung disease caused by the inhalation of crystalline silica and is characterized by chronic leukocyte infiltration, fibroblast proliferation, and excessive collagen deposition resulting in the formation of localized silicotic granulomas in the lung [1]. Silicosis reduces lung functions and still remains a prevalent health problem throughout the world, particularly in developing nations [2], [3].

It is generally accepted that silica particles activate innate immunity, culminating in the release of pro-inflammatory mediators and growth factors for fibroblasts, which are crucial in driving alveolitis, lung fibrosis, and possibly carcinogenesis [4], [5]. Innate immune responses to silica require particle interactions with Fc and MARCO receptors on macrophages or dendritic cells [6]–[8]. In response to silica, the processing and secretion of the pro-inflammatory cytokine Interleukin-1 β (IL-1β) in a caspase-1/ NOD-like receptor family pyrin domain containing 3 (NLRP3) inflammasome-dependent manner initiates a cascade of innate immune responses leading to neutrophilic inflammation and granulomas [9]–[13]. Tumor Necrosis Factor α (TNF-α), which promotes the activation of innate immune cells, is also implicated in the pathogenesis of silicosis. Silica-induced lung inflammation and granuloma formation are reduced by the administration of TNF inhibitors or in animals deficient in TNF-α receptors [14]–[16]. The exacerbation of innate immune responses by repeated LPS exposure also amplifies the granulomatous response to silica in mice [17]. Finally, systemic inhibition of NF-kappa B activation with a pharmacological inhibitor decreases the severity of experimental silicosis [18]. Clinical observations have also led to the assumption that the activation of innate immunity is an important orchestrator of the silicotic process. In humans, several investigators have stressed the close link between innate immune cytokines (IL-1 and TNF-α), chronic inflammation and silicosis [14], [19], [20]. In conclusion, the prevailing pathogenic paradigm states that silicotic granuloma formation is dependent on the activation of innate immunity.

MyD88 links members of the toll-like receptor (TLR), nucleotide-binding oligomerization domain receptor (NLR) and interleukin-1 receptor (IL-1R) superfamily to the downstream activation of NF-kappa B and mitogen-activated protein kinases [21]. To better characterize the innate immune signals involved in the development of particle-induced inflammation, granuloma formation and fibrosis, we determined lung responses to silica in mice KO for MyD88. We demonstrated that MyD88 is crucial for the development of silicotic inflammation and granulomas. However, MyD88-KO mice developed pronounced lung fibrosis even in the absence of progressive inflammation indicating that fibrogenesis is a pathological process, which can also occur independently of inflammatory and innate immune responses.

## Methods

### Animals

C57BL/6 mice were purchased from Charles River Laboratory (Brussels, Belgium). MyD88-, ASC-, IL-1R1-, IL-1α-, IL-1β-, NALP3-, TLR2/4, TLR3-, TRIF-, IL-23p19-, TCRδ- [22]–[28] deficient mice (all on a C57BL/6 background) were obtained from Transgenose (Orleans, France). Mice were maintained in sterile microisolators with sterile rodent feed and acidified water and housed in positive-pressure air-conditioned units (25°C, 50% relative humidity) on a 12-h light/dark cycle. The experimental protocol was approved by the local ethical committee for animal research at the Université catholique de Louvain (2010/UCL/MD/034) and conformed to the Belgian and European Community regulations (LA1230312 and CEE n° 86/609).

### Animal treatments

To allow sterilization and inactivation of any trace of endotoxins, silica particles (DQ12; SiO_2_, d50 = 2.2 µm) were heated at 200°C for 2 h immediately before suspension in sterile and pyrogenic 0.9% saline and administration. The suspension of silica (2.5 mg in 50 µl of saline per mouse) was injected directly into the lungs by pharyngeal aspiration.

### Bronchoalveolar lavage (BAL) and whole lung homogenates

The mice were sacrificed with an i.p. injection of 20 mg sodium pentobarbital (Certa, Braine-l'Alleud, Belgium) at selected time intervals post silica treatment. Bronchoalveolar lavage (BAL) was performed by cannulating the trachea and infusing the lungs 4 times with 1.5 ml of 0.9% saline. The BAL fluid (BALF) was centrifuged (280 g, 4°C, 10 min.) and the cell-free supernatant was used for biochemical measurements. The cell pellets of the BAL fractions were resuspended in saline to determine cell numbers on a Burker cell chamber and cell differentials on cytocentrifuge preparations stained with Diff-Quick (Baxter, Lessines, Belgium). Lavaged lungs were then perfused via the right heart ventricle with saline, excised and placed into a Falcon tube chilled on ice for gene expression (1 left lobe) and collagen assays (3 right lobes). For this last analysis, two ml of 0.9% saline were added and the lungs were homogenized with an Ultra-Turrax T25 homogenizer (Janke and Kunkel, Brussels, Belgium) for 30 seconds and stored at −80°C for later use. Lung collagen accumulation was estimated by measuring the hydroxyproline (OH-proline) contents by HPLC in lung homogenates.

### Flow cytometry analysis and CD4^+^ T lymphocyte purification

Unlavaged lungs from WT and MyD88 KO mice were perfused excised and washed in HBSS. The 3 right lobes of the lungs were cut into small pieces and digested enzymatically for 45 min at 37°C. The digestion buffer was made of Liberase CI (now replaced by Liberase TL) (5 mg/lung) (Roche Diagnostics, Meylan, France) and DNAse (250 µg/lung) (Worthington Biochemical Corp., Lakewood, NJ, USA) dissolved in HBSS (9.6 ml/lung) (Invitrogen) and supplemented with 1% of antibiotics (100 U/ml penicillin, 100 µg/ml streptomycin and 0.25 µg/ml fungizone; Invitrogen). The resulting cell suspension was filtered through a 70 µm filter (BD Biosciences, Bedford, MA, USA). After centrifugation (280 g, 10 min, 4°C), aliquots of cell suspensions were used to determine total cell numbers. Fluorescent labelling of lung cell suspensions was undertaken upon re-suspension in HCF medium (Hanks medium for flow cytometry) with 3% decomplemented fetal bovine serum (FBS, Invitrogen). Fc receptors were blocked with anti-CD16/32 (clone 2.4G2, BD Biosciences). Cells were stained using antibodies specific for CD4 (clone RM4-5) and CD8 (clone 53–6.7) obtained from BD Biosciences. Samples were fixed in a 1.25% paraformaldehyde solution in PBS for at least 1 h, acquired on a FACSCalibur (BD Biosciences) and analyzed using the CellQuest software (BD Biosciences). Analysis of cell populations was undertaken with appropriate gating according to side and forward scatters to exclude dead cells as well as silica particles. CD4+ T cells were separated and isolated by magnetic cell separation system (MACS, Miltenyi Biotec, Bergisch Gladbach, Germany) by positive selection with anti-CD4 magnetic beads. The purity of the obtained immune cell preparations was routinely >95% as assessed by Diff-Quick staining or FACS analysis (CD5+ positive lymphocytes).

### RNA extraction and quantification

Perfused and lavaged left lobe was homogenized on ice in 2 ml of Tripure reagent (Roche) using an Ultra-Turrax T25. Total RNA extraction was performed according to Tripure manufacturer's instructions. 1 µg of RNA was reverse transcribed with M-MLV Reverse Transcriptase (Invitrogen) with 350 pmol random hexamers (Eurogentec, Seraing, Belgium). Resulting cDNA was then diluted 10 or 50 fold and used as template in subsequent polymerase chain reactions (PCR). Sequences of interest were amplified using the following forward primers (Invitrogen): 5′-CGG CTA CCA CAT CCA AGG AA-3′ (18S RNA), 5′-CCC AGG AAA GAC AGC AAC CTT-3′ (Foxp3), 5′-TTC CTG AGG AAC TGT ATG AAA TGC-3′ (PDGF-B), 5′-CCC CAC TGA TAC GCC TGA GT-3′ (TGF-β_1_), 5′-GCT CCA GAA GGC CCT CAG A-3′ (IL-17A) and 5'-TCC CGA GAT GCT GTC AAG TTT-3' (RORγ) and reverse primers: 5′-ATA CGC TAT TGG AGC TGG AAT TAC C-3′ (18S RNA), 5′-CGC CAT CTT CCC AGC CAG GTG-3′ (Foxp3), 5′-TCA GCC CCA TCT TCA TCT ACG-3′ (PDGF-B), 5′-AGC AGT GAG CGC TGA ATC G-3′ (TGF-β1), 5′-AGC TTT CCC TCC GCA TTG A-3′ (IL-17A) and 5'-AGT GTA TGT AAG TGT GTC TGC TCC G-3' (RORγ). The expression of the different genes was normalized on that of β-actin or 18S RNA. PCR was performed with AmpliTaq Gold polymerase (Invitrogen) according to manufacturer's instructions with the following temperature program: 10 min 95°C, (15 s 95°C, 1 min 60°C) ×40 cycles, 5 min 4°C. Amplified DNA fragments were purified from a 2% agarose gel with the “NucleoSpin Extract II” kit (Macherey-Nagel, Düren, Germany) and then serially diluted to serve as standards in quantitative real-time PCR (RT-qPCR). Reverse transcribed mRNAs were finally quantified by RT-qPCR using SYBR Green technology on an ABI Prism 7000 Sequence Detection System (Applied Biosystems, Foster City, USA). The SYBR Green was used according to the following program: 2 min 50°C, 10 min 95°C, (15 s 95°C, 1 min 60°C) ×40 cycles. In order to verify specific amplification, a dissociation curve was obtained by increasing temperature up to 95°C during 20 minutes. Five µl of diluted cDNA or standards were amplified with 300 nM of the described primers using SYBR Green PCR Master Mix (Applied Biosystems) in a total volume of 25 µl. RT-qPCR for mouse IL-10 was performed with qPCR Mastermix TaqMan following the manufacturer's protocols (Invitrogen).

### Enzyme-Linked Immunosorbent Assays (ELISA)

Mouse IL-1β and PDGF-BB concentrations in BAL fluid were measured with ELISA kits (R&D Systems, Minneapolis, MN) following the manufacturer's protocols. The detection limit of these ELISA was 2 pg/ml.

### Histology and immunohistochemistry

The unlavaged left lobe of the lungs was excised and fixed in a 3.65% formaldehyde solution (Sigma-Aldrich) for histopathology studies. After overnight fixation, lung pieces were embedded in paraffin, 5 µm thick sections were taken and stained with hematoxylin and eosin or impregnated with silver according to Gordon and Sweets [29]. Histological sections stained with Masson's trichrome were analyzed in a blinded manner by an independent pathologist on a Jenamed microscope at a 100-fold magnification to quantify fibrotic nodules and diffuse areas of fibrous thickening of alveolar septa. The number of discrete fibrotic nodules, of areas showing diffuse fibrous thickening of alveolar septa and of lymphoid aggregates were counted in each microscopic field and counts were added to cover the whole histological section. A single histological section of lung was analyzed per mouse.

### Statistical analysis

For comparison among multiple groups, data were evaluated by one-way analysis of variance (ANOVA) using the Student Neuman-Keul's test when appropriate. For comparison between two groups, Student's unpaired t-tests were performed. In both cases, significance was considered at p<0.05 (two-tailed).

## Results

**Figure 1:**
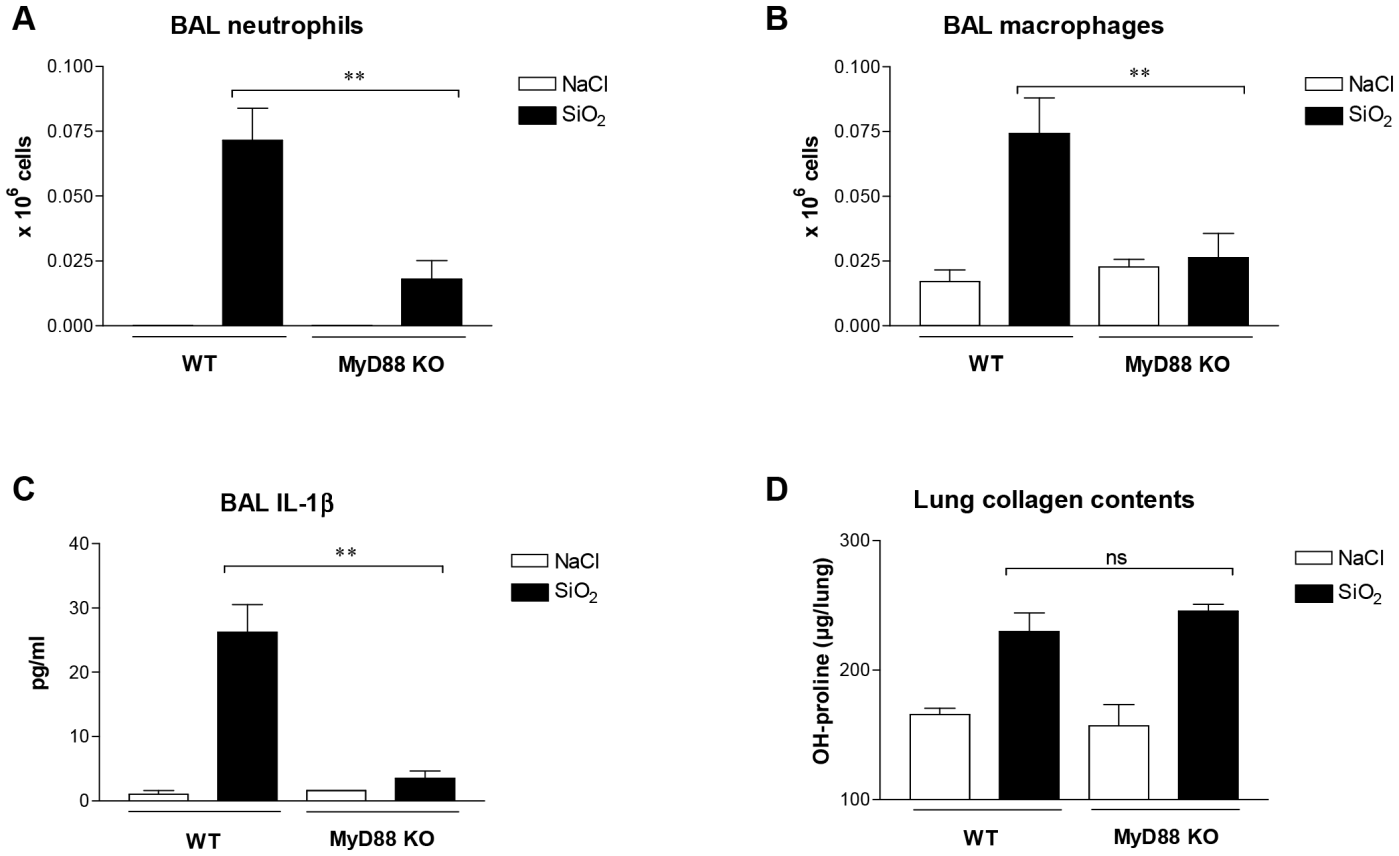
Lung inflammation but not collagen accumulation is reduced in MyD88-KO mice treated with silica particles. Neutrophil (**A**) and macrophage (**B**) numbers in the bronchoalveolar lavage (BAL) fluid of wild-type (WT) and MyD88-knockout (MyD88-KO) mice treated with silica (SiO_2_, 2.5 mg/mouse) or saline solution (NaCl) and sacrificed at day 60. Quantification of IL-1β (**C**) by ELISA in the BAL fluid of WT and MyD88-KO mice treated with SiO_2_ or NaCl at day 60. Lung fibrosis assessed by OH-proline contents (**D**) in WT and MyD88-KO mice after silica or saline instillation (d60). Bars represent means ± SEM (n = 4–6). These results were treated statistically by a t- test. ns indicates no statistically significant difference and ** = p<0.01 indicate statistically significant difference between values measured in silica-treated WT and silica-treated KO mice.

The MyD88 adaptor protein is well known to play an essential role in innate immune responses [21]. To determine whether MyD88 is involved in silica-induced lung inflammation and fibrosis, we assessed long-term (day 60) lung responses after crystalline silica particle treatment (SiO_2_, 2.5 mg/mouse) in MyD88-knockout (KO) and wild-type (WT) mice. Bronchoalveolar lavage (BAL) neutrophil numbers were significantly reduced after silica treatment in MyD88-KO mice in comparison with WT animals (Figure 1 A). Accumulation of BAL macrophages was also decreased in absence of MyD88 (Figure 1 B). Expression of the dominant pro-inflammatory cytokine IL-1β was significantly reduced in KO mice after silica particles (Figure 1 C). The reduction of lung inflammation in absence of MyD88 was not associated with a corresponding limitation of fibrogenesis, however. Indeed, lung OH-proline contents were similarly increased in silica-treated KO and WT mice (Figure 1 D).

**Figure 2:**
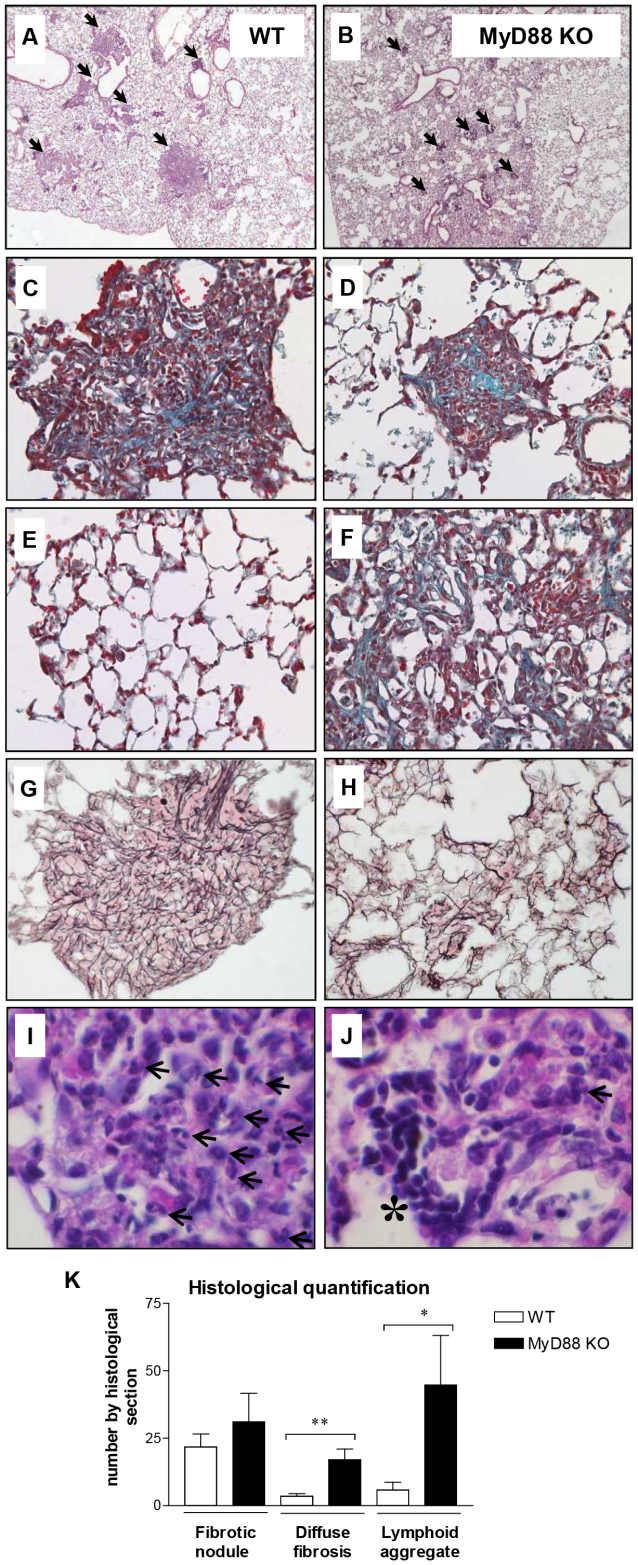
MyD88-KO mice treated with silica particles developed limited granuloma and neutrophil infiltration but marked fibrosis and lymphocyte accumulation. 5-µm sections of paraffin-embedded lung tissue of wild-type (WT) and MyD88- knockout (MyD88-KO) mice treated with silica (SiO_2_, day 60) were stained with hematoxylin and eosin (panels **A–B** and **I–J**; neutrophils are indicated by black arrows and lymphocyte aggregates by an asterisk), with Masson's trichrome (panels **C–F**) or with silver (panels **G–H**). Sections are representative of 3–4 mice examined. Magnification 50X (panels A and B), 100X (panels **C–H**) and 400X (panels I–J). Histological quantification of fibrotic nodule, diffuse fibrosis and lymphoid aggregate numbers (**K**). These results were treated statistically by a t- test. * = p<0.05, ** = p<0.01 indicate statistically significant difference between values measured in silica-treated WT and silica-treated KO mice.

Qualitative and quantitative histopathological analyses were conducted to clarify these observations (Figure 2 A–J and K, respectively). WT mice developed localized neutrophilic granulomas (Figure 2 A, C and I) while in MyD88-KO animals, granulomatous nodules were less developed and contained less granulocytes (Figure 2 B, D and J). While fibrosis was mainly localized in granulomas in WT animals (Figure 2 G and K), the pulmonary parenchyma of silica-treated MyD88-KO mice showed significant zones of less organized fibrosis (Figure 2 F, H and K). Altogether, these results emphasized that the MyD88 axis is important for neutrophilic inflammation and granuloma formation, but apparently not for the fibrotic response.

**Figure 3:**
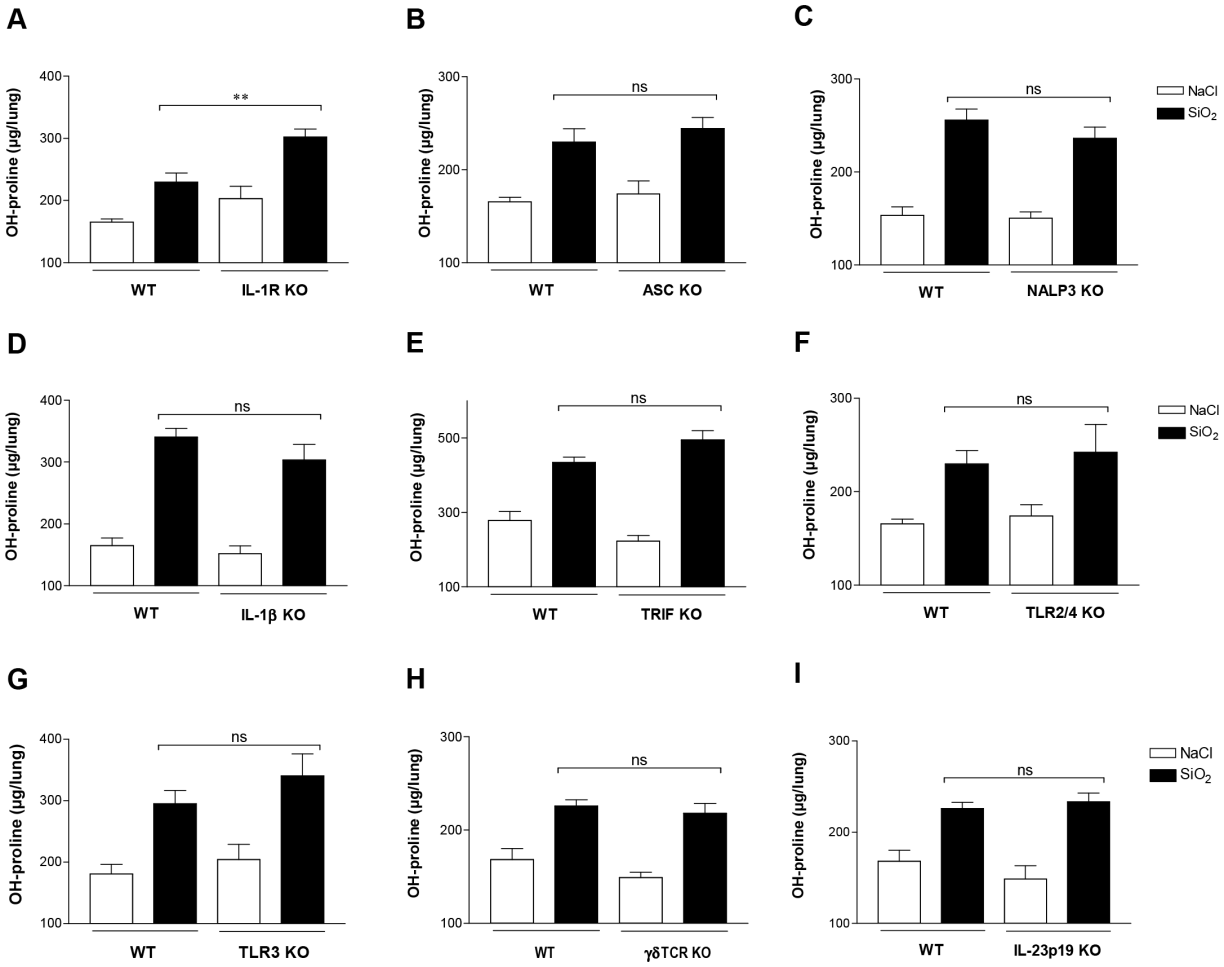
Robust fibrotic response in the absence of innate immune receptors or ligands. Hydroxyproline (OH-proline) content assessed in lung homogenates of mice 60 days after saline (NaCl) or silica (SiO_2_) instillation in wild-type (WT), IL-1R- (**A**), ASC- (**B**), NALP3- (**C**), IL-1β- (**D**), TRIF- (**E**), TLR2/4- (**F**), TLR3- (**G**), γδTCR- (**H**), IL-23p19- (**I**) knockout (KO) mice. Bars represent means ± SEM (n = 3–5). These results were treated statistically by a t- test. ns indicates no statistically significant difference and ** = p<0.01 indicates statistically significant difference between values measured in silica-treated WT and silica-treated KO mice.

To strengthen the conclusion that the lung fibrotic lesions induced by silica occur despite the limitation of MyD88-related inflammation, we additionally studied mice deficient for other key innate immune mediators. Recently, it has been shown that the NALP3-inflammasome/IL-1 axis plays a key role in pulmonary innate immune responses to silica [11]. We thus tested whether this pathway is implicated in silica-induced lung inflammatory and fibrotic responses. As observed in MyD88-KO animals, we noted that genetic deficiency in IL-1R was associated with marked reduction of BAL neutrophil and macrophage accumulation (Figure S1 A and B) as well as limited granuloma formation after silica (Figure S1 C and D). However, sustained fibrosis in IL-1R-KO mice was observed by assessing lung collagen contents (Figure 3A). Histological analyses revealed the presence of diffuse fibrosis distributed in the parenchyma of silica-treated IL1R-KO mice (Figure S1 E). Limited chronic lung granulomatous inflammation with reduced neutrophilic and macrophages infiltration was also observed in absence of ASC, NALP3 and IL-1β (data not shown). However, lung collagen accumulation after silica was not reduced in ASC-, NALP3-, and IL-1β-KO mice compared to their corresponding WT mice at day 60 (Figure 3 B–D). Similar observations were made by studying IL-1α-, IL-33R- and IL-18R-KO mice (not shown). In conclusions, less organized lung fibrosis was observed in these MyD88/IL-1 family-related models.

We then examined whether alternative inflammatory pathways may contribute to explain fibrotic response in MyD88-KO mice. TLR signaling and the resulting transcriptional activation of innate immune- and inflammatory-related genes require the adaptor MyD88, except for TLR3 and TLR4 signaling, which need another adaptor protein, TRIF [30]. We found that TLR3, TLR4 and TRIF are not crucial in silica-induced chronic lung granulomatous inflammation and fibrosis. Indeed, at day 60 after silica, the levels of neutrophil and macrophage inflammation (not shown) and collagen accumulation (Figure 3 E, F and G) were not different between TRIF-, TLR3-, TLR2/4-KO and WT mice. Finally, γδ T lymphocytes and their necessary cytokine IL-23 are additional components of pulmonary innate immunity [31]. They did not appear involved in silica-induced lung granulomatous inflammation and fibrosis since lung neutrophil and macrophage accumulation (not shown) and collagen deposition (Figure 3 H and I) were similar in γδ TCR- and IL-23p19-deficient mice compared with WT animals 60 days after silica. Altogether, these data clearly strengthened the observation that lung fibrosis can occur in absence of innate immunity activation.

**Figure 4:**
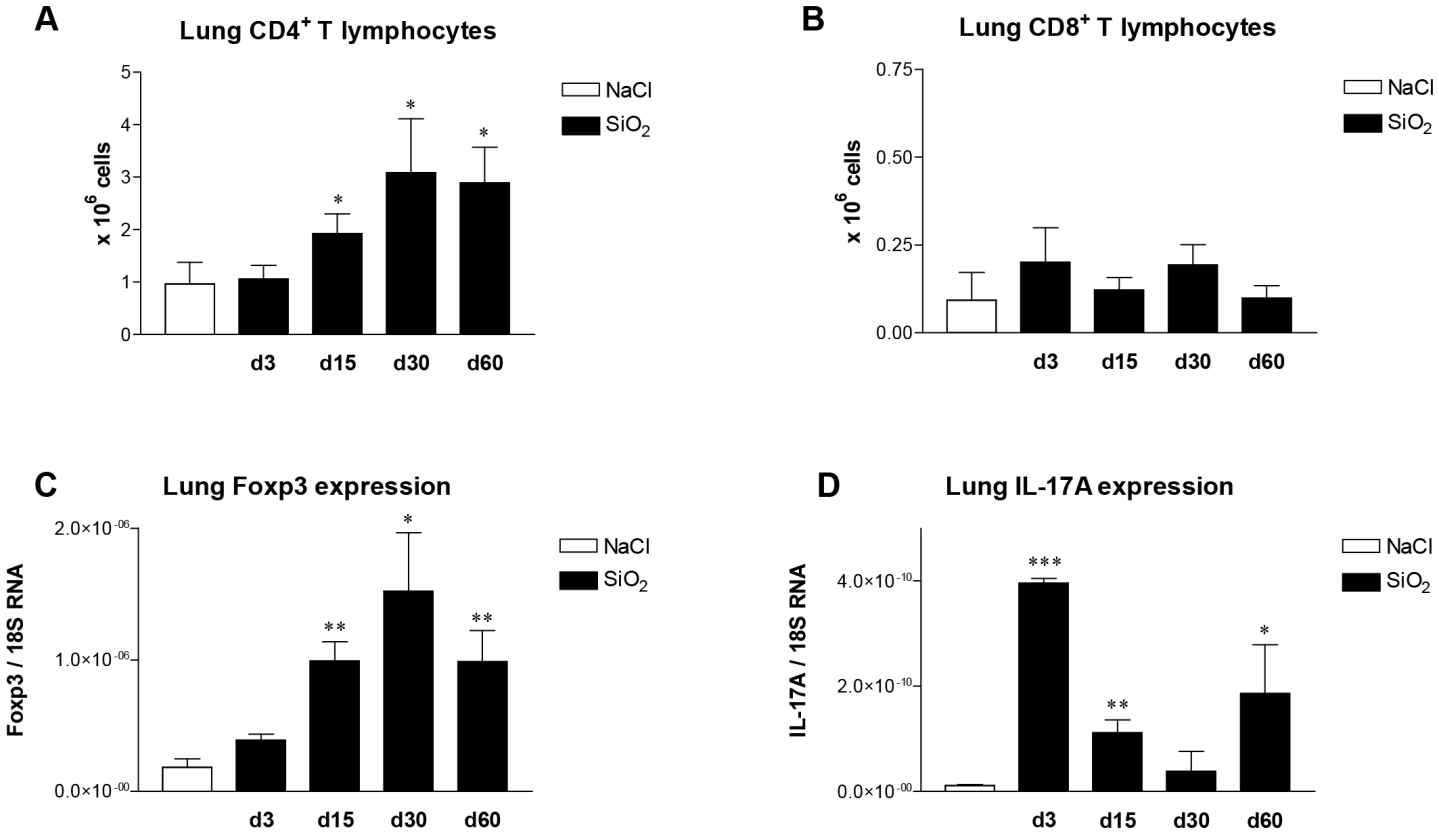
Pulmonary accumulation of T regs and Th17 lymphocytes is associated with the development of silica-induced lung fibrosis. Number of CD4+ (**A**) and CD8+ (**B**) analyzed by flow cytometry in lung cell suspensions obtained from wild-type mice after silica (SiO_2_, d3 to d60) or saline (d0) instillation. Expression of Foxp3 (**C**) and IL-17A (**D**) analyzed by RTqPCR in the lungs obtained from wild-type mice after silica (d3 to d60) or saline (d0) treatment. Expression of Foxp3 and IL-17A was normalized on that of 18S RNA. Bars represent means ± SEM (n = 3–5). These results were treated statistically by a Student Neuman-Keul's test. * = p<0.05, ** = p<0.01, *** = p<0.001 indicate statistically significant difference between values measured in saline-treated mice and silica-treated mice.

To clarify the mechanisms governing silica-induced fibrosis in absence of MyD88-mediated innate immune signals, we further detailed the lung responses to silica in MyD88-KO mice. Lung sections revealed the concomitant presence of lymphoid aggregates with fibrosis in these animals (Figure 2 J and K). Considerable attention has been paid to determine the role of T lymphocytes in fibrosis [32]. In particular, it has been shown that immunosuppressive Foxp3-expressing regulatory T lymphocytes (T regs) [33] and inflammatory IL-17A-producing T lymphocytes (Th17) [34] can drive a pulmonary fibrotic process in humans and animals. In accordance with these observations, we noted that a recruitment of lung CD4^+^ T lymphocytes (Figure 4 A), T regs (Figure 4 C) and Th17 cells (IL-17A, Figure 4 D), but not CD8^+^ T (Figure 4 B) or NK cells (not shown), was associated with lung fibrosis in WT animals after silica treatment. The expression of RORγ, a specific transcription factor of Th17 cells, was not significantly induced in lung tissue after silica treatment (not shown). No Th1 or Th2 polarization was observed during fibrogenesis and the lung expression of T-bet, IFN-γ, IL-4, IL-13 and GATA3 was not modified by silica (not shown).

**Figure 5:**
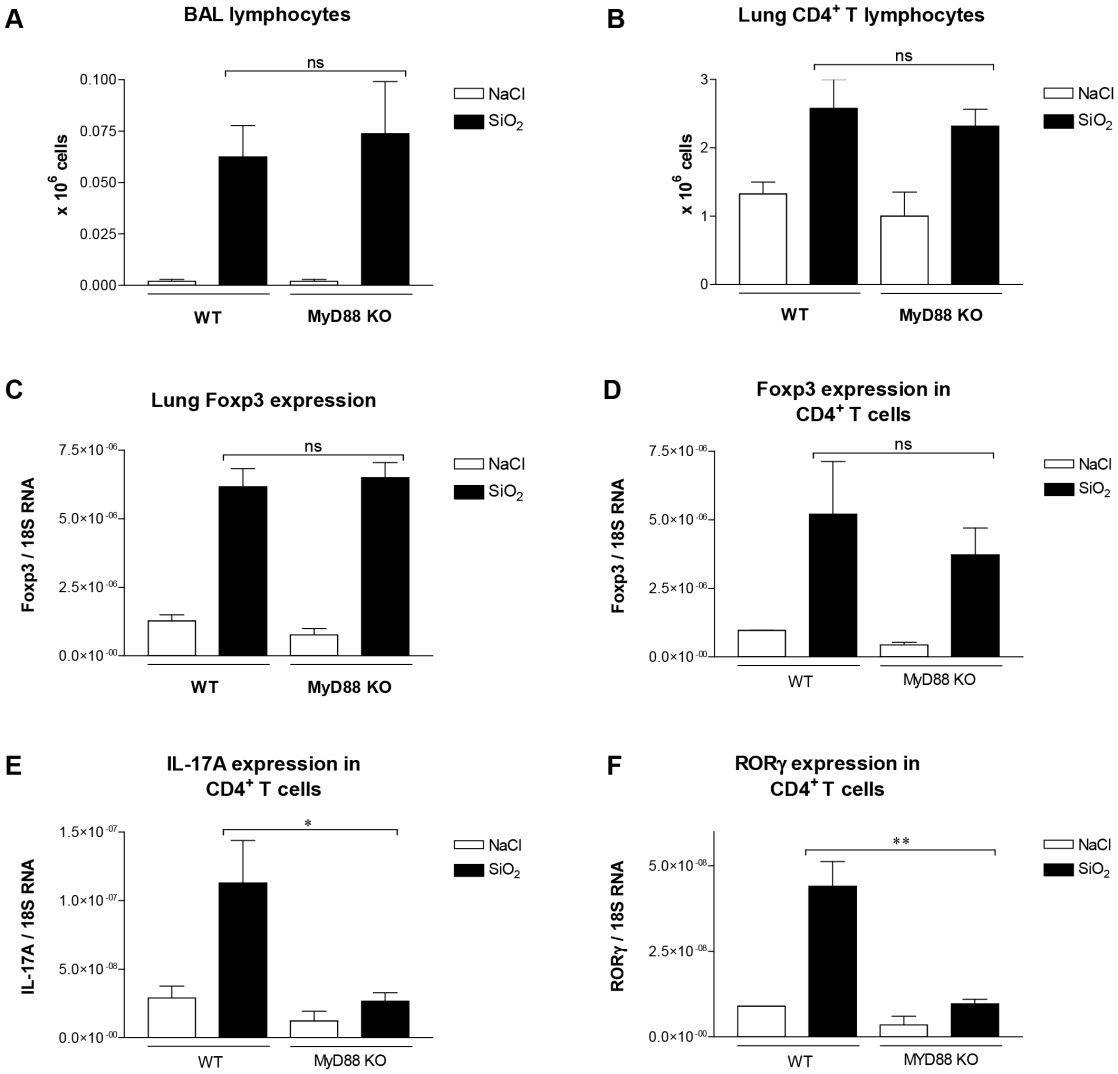
Lung accumulation of Th 17 cells but not T regs is limited in the absence of MyD88 signalling. Lymphocyte numbers in the bronchoalveolar lavage (BAL) fluid (**A**) and CD4+ T lymphocyte numbers in lung suspensions (**B**) obtained from wild-type (WT) and MyD88-knockout (MyD88-KO) mice 60 days after silica (SiO_2_) or saline solution (NaCl) treatment. Expression of Foxp3 (**C** and **D**), IL-17A (**E**) and RORγ (**F**) analyzed by RTqPCR in lungs (**C**) and in lung CD4+ T lymphocytes (**D–F**) obtained from WT and MyD88-KO mice 60 days after silica or saline treatment. Expression of Foxp3 and IL-17A was normalized on that of 18S RNA. Bars represent means ± SEM (n = 3–5). These results were treated statistically by a t-test. ns indicates no statistically significant difference and * = p<0.05 and ** = p<0.01 indicates statistically significant difference between values measured in silica-treated WT mice and silica-treated KO mice.

We next focused on the expansion of CD4^+^ T, T regs and Th17 lymphocytes during the fibrotic process in silica-treated MyD88-KO mice. Contrary to neutrophils and macrophages (Figure 1 A and B), BAL lymphocyte and lung CD4^+^ T cell numbers were not reduced in MyD88-KO mice compared to WT animals 60 days after silica (Figure 5 A and B). Foxp3 expression in lung tissue or in purified pulmonary CD4^+^ T lymphocytes was similar in MyD88-KO and WT mice (Figure 5 C and D). In contrast, IL-17A expression in CD4^+^ T lymphocytes and lungs (Figure 5 E and not shown) as well as RORγ expression in CD4^+^ T lymphocytes (Figure 5 F) were decreased in absence of MyD88, revealing that the pulmonary influx of Th17 lymphocytes induced by silica was mediated by MyD88-dependent innate immunity. Altogether, these results indicated that the development of lung fibrosis in MyD88-KO mice is rather associated with the presence of immunosuppressive T regs than with inflammatory Th17 lymphocytes.

**Figure 6:**
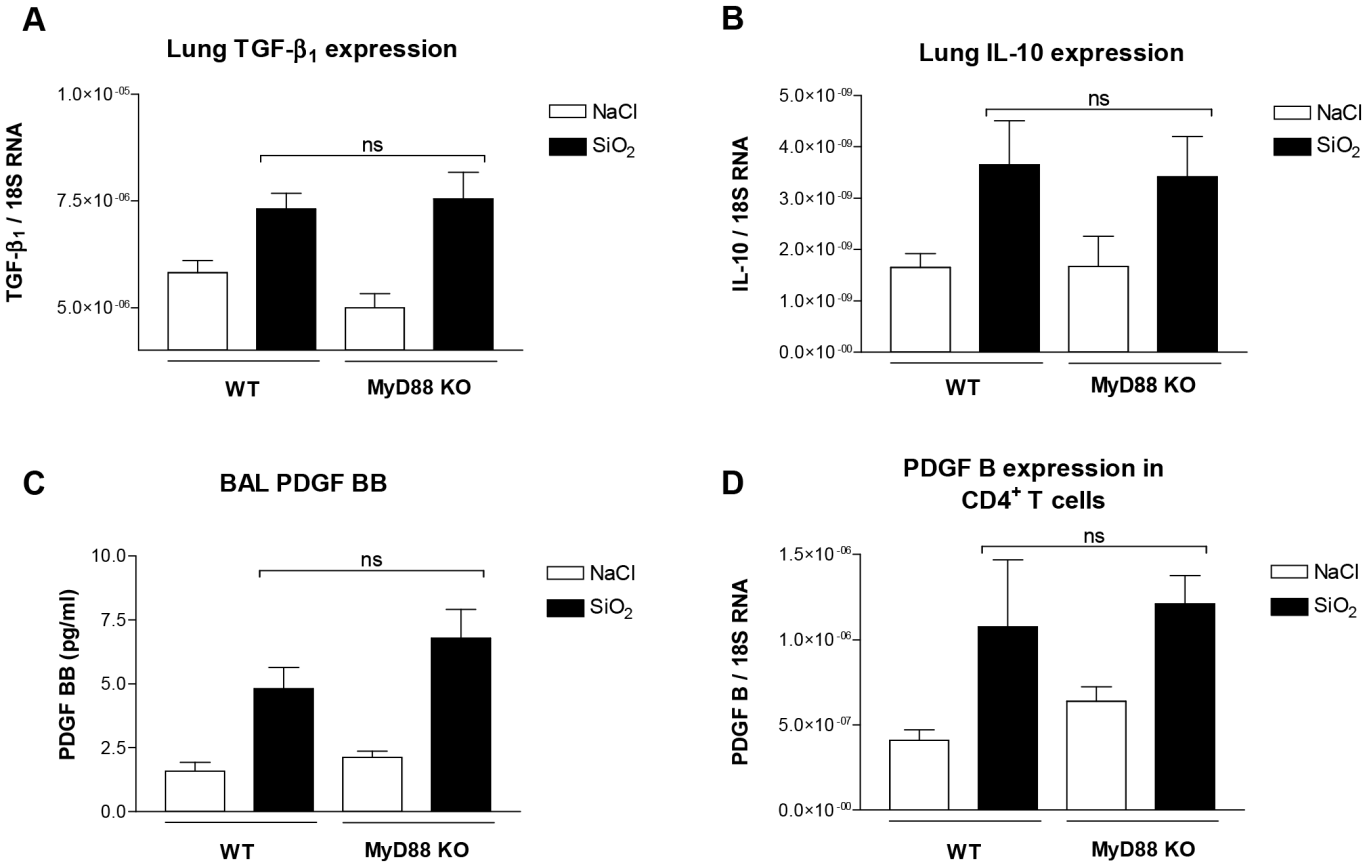
Lung expression of TGF-β1, IL-10 and PDGF-B is not reduced in MyD88-KO mice treated with silica particles. Lung expression of TGF-β1 (**A**), IL-10 (**B**) and PDGF-BB (**C**) analyzed by RTqPCR in tissue or by ELISA in BAL of wild-type (WT) and MyD88-knockout (MyD88-KO) mice 60 days after silica (SiO_2_) or saline (NaCl) treatment. Expression of PDGF B (**D**) analyzed by RTqPCR in lung CD4+ T lymphocytes purified from NaCl- or SiO_2_-treated WT and MyD88-KO mice at day 60. Expression of TGF-β1, IL-10 and PDGF-B was normalized on that of 18S RNA. Bars represent means ± SEM (n = 3–5). These results were treated statistically by a t- test and no statistically significant difference (ns) between values measured in silica-treated WT mice and silica-treated KO mice was noted.

To further compare the responses that may contribute to explain the development of fibrosis in MyD88-KO mice, we measured the pulmonary expression of the immunosuppressive and pro-fibrotic cytokines Transforming Growth Factor-β (TGF-β) and IL-10. Sixty days after silica, the levels of TGF-β and IL-10 transcripts were similarly up-regulated in MyD88-KO and in WT mice (Figure 6 A and B). We also assessed the expression of Platelet-Derived Growth Factor B (PDGF-B), which is produced by the T reg subpopulation and operative in experimental silicosis [33]. First, PDGF-BB was released at the same level in BAL fluid of MyD88-KO and WT mice after silica exposure (Figure 6 C). In addition, CD4^+^ T cells purified from silica-treated MyD88-KO and WT mice similarly expressed PDGF-B (Figure 6 D). Thus, T regs are similarly accumulated and similarly express PDGF-B during the fibrotic process in MyD88-KO and WT mice.

## Discussion

Our study demonstrates that the MyD88-signaling pathway mobilized during the lung responses to silica particles organizes granuloma formation, Th17-associated inflammatory responses and neutrophil accumulation; three major features of silicosis. These observations are in accordance with studies showing that inflammatory Th lymphocytes or IL-1-related responses are decisive in the development of neutrophilic granulomatous responses in experimental silicosis [12], [35]–[37]. Interestingly, this crucial pathway has also been implicated in acute or chronic lung inflammatory diseases experimentally induced by cigarette smoke, bleomycin, inorganic dusts or elastase [38]–[41]. This large body of evidence strongly suggests that MyD88 represents a crucial mediator in the establishment of lung inflammation and an appropriate target for down regulating deleterious inflammatory reactions in diverse conditions.

While there is evidence showing that MyD88-associated innate immunity is also necessary in scar formation in models of lung fibrosis induced by bleomycin or elastase [39], [41], controversies and uncertainties still exist concerning the exact role of innate immune responses in pulmonary fibrosis induced by silica in mice. Indeed, experimental data indicate that key innate immune mediators (i.e. IL-12, TNF-α and type I interferons) and cells (i.e. DC and macrophages) are dispensable for the development of lung fibrosis in silica-treated mice [42]–[48]. In addition, recent observations raise the possibility that innate immune signals may negatively regulate the development of experimental fibrosis. Indeed, inhibition of the NF-kappa B cascade aggravated the outcome of silica-induced lung fibrosis [18] and TLR4 pathway activation reduced the amplitude of pulmonary fibrosis in mice exposed to silica [49]. Observations in this study also support the view that innate immunity is not absolutely required for the development of the lung fibrotic response to silica. Indeed, abnormal accumulation of collagen during experimental silicosis was not reduced by abrogating the main signal transduction pathways activated by TLRs. In addition, the fact that silica-induced lung collagen deposition was not quantitatively reduced in the absence of several key members of the IL-1 family (ASC, NALP3, IL-1R, IL-18R, IL-33R, IL-1 α and β) strongly argues for a minor role of NLR- and inflammasome-mediated innate immunity in pulmonary fibrogenesis after silica. Finally, several elements of the recent literature also support the paradigm of a fibrotic reaction to silica developing uncoupled from inflammation. Indeed, it has been observed that the innate immunity Aryl hydrocarbon receptor (AhR) regulates silica-induced inflammation and granuloma formation without affecting the fibrotic process [50]. Similarly, type I interferons are necessary in the establishment of silica-induced lung inflammation while they are not necessary for lung fibrosis [42]. Finally, different treatments with anti-inflammatory molecules reduced lung inflammation without modifying collagen deposition in animals [51], [52]. Conventional anti-inflammatory agents do not alter silicosis and final outcome in patients and there is no evidence that corticosteroid or prednisolone confer significant benefits for patients with chronic or accelerated silicosis [1], [53].

Taken together, these observations therefore allude to the existence of alternative pathological pathways as drivers of chronic fibrosis in particle-related lung disorders. In this context, it has been shown that silica-induced lung fibrosis is accompanied in mice by a progressive immunosuppressive process and results from the action of the immunosuppressive cytokines TGF-β and IL-10 produced to limit the development of chronic inflammatory and innate immune responses [54]–[61]. Recent results also indicated that regulatory T cells CD4+ Foxp3+ (T regs) accumulated during the development of pulmonary fibrosis, possess a strong fibrotic activity in mice treated with silica [33], [62]. The findings of the present study are consistent with the idea that immunosuppressive cells (T regs) and their related cytokines (TGF-β, IL-10 and PDGF) may contribute to explain the development of the lung fibrotic responses induced by silica also when innate immune responses are absent. In WT mice, the fibrotic response was localized in granulomas where T regs accumulated, whereas it was less organized in MyD88-KO mice in the absence of a robust granuloma reaction. Our study furthermore demonstrates that T reg-related responses are independent of inflammation and innate immunity. It remains, however, important to determine whether immunosuppression is also operative in humans exposed to silica who develop lung fibrosis. This possibility could explain, at least in part, the well described increased risk of infection and tuberculosis in patients with silicosis [1].In this study, we did not formally demonstrate that fibrosis in absence of MyD88 only results from T reg-related pro-fibrotic activities. Indeed, we cannot exclude that other populations of lymphocytes such as B and CD8+ T cells, potentially present in the lymphoid aggregates, could also participate in the pathological process [63], [64].

New evidence indicates that Th17 cells play an important pathogenic role in granuloma development in lung and liver [65]–[67]. Th17 pathway promotes tissue granulomatous inflammation through the activation of pro-inflammatory cytokine/chemokine release and the recruitment of neutrophils. In this study, we observed an association between the dysregulated granuloma formation in MyD88 mice and the limited accumulation of Th17-related cells and neutrophils. Altogether, these observations support Th17 cell involvement in granulomatous inflammation after silica exposure. Human sarcoidosis has a remarkable propensity for spontaneous resolution and this correlates with the marked presence inside the granuloma of the interleukin-1 receptor antagonist (IL-1RA), an endogenous inhibitor of IL-1 bioactivity [68], [69]. We also observed limited granuloma development in IL-1-related deficient mice treated with silica as in MyD-88 KO animals. Interestingly, it is well demonstrated that IL-1 strongly amplifies IL-17 production and Th17 polarization [70]. Our data support a link between MyD88, IL-1 and Th17 responses during the development of granulomas. Therefore, the neutralization of this particular cytokine axis represents an attractive therapeutic possibility to reduce lung granulomatous disorders induced by particles.

In conclusion, our observations suggest that inflammation and fibrosis in silicosis represent two distinct processes requiring the activation of two different immune pathways. Silica-induced pulmonary inflammatory and granulomatous responses are related to MyD88-dependent innate immunity while lung fibrosis is uncoupled from this pathway and linked to T reg-related immunosuppression. This new concept could contribute to better identify, manage and treat silicotic patients.

## Supporting Information

Figure S1 **IL-1R-KO mice treated with silica particles developed limited granuloma and macrophage and neutrophil infiltration but diffuse fibrosis.** Neutrophil (A) and macrophage (B) numbers in the bronchoalveolar lavage (BAL) fluid of wild-type (WT) and IL1R-knockout (IL-1R-KO) mice treated with silica (SiO_2_, 2.5 mg/mouse) or saline solution (NaCl) and sacrificed at day 60. Bars represent means ± SEM (n = 4–6). These results were treated statistically by a t- test. ns indicates no statistically significant difference and ** = p<0.01 indicate statistically significant difference between values measured in silica-treated WT and silica-treated KO mice. 5-µm sections of paraffin-embedded lung tissue of wild-type (WT) and IL-1R-KO mice treated with silica were stained with hematoxylin and eosin (C, 50X) or with Masson's trichrome (panels D and E, 200X). Sections are representative of 3-4 mice examined.
(PDF)
