# Correction: Uncoupling between Inflammatory and Fibrotic Responses to Silica: Evidence from MyD88 Knockout Mice

**DOI:** 10.1371/journal.pone.0116134

**Published:** 2014-12-16

**Authors:** The PLOS ONE Staff

Dr. Giulia Giordano should be included in the author byline. She should be listed as the second author, and her affiliation is 1: Louvain centre for Toxicology and Applied Pharmacology (LTAP), Institut de Recherche Expérimentale et Clinique (IREC), Université catholique de Louvain, Brussels, Belgium.

The correct citation is: Lo Re S, Giordano G, Yakoub Y, Devosse R, Uwambayinema F, et al. (2014) Uncoupling between Inflammatory and Fibrotic Responses to Silica: Evidence from MyD88 Knockout Mice. PLoS ONE 9(7): e99383. doi:10.1371/journal.pone.0099383
