# Supplementary figures and images for: Uncoupling between Inflammatory and Fibrotic Responses to Silica: Evidence from MyD88 Knockout Mice
Source: PLoS One. 2014 Jul 22;9(7):e99383. doi: 10.1371/journal.pone.0099383 (PMC4106757; doi:10.1371/journal.pone.0099383)

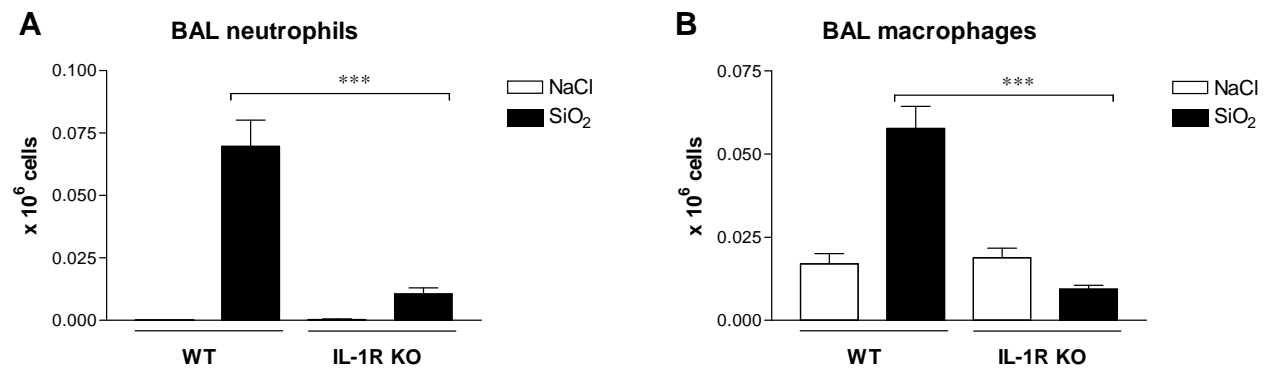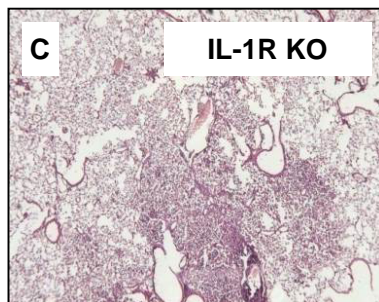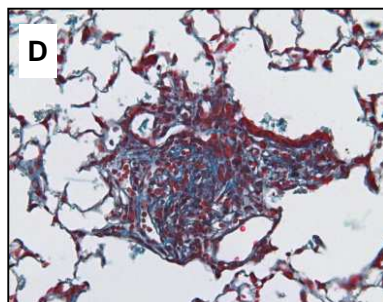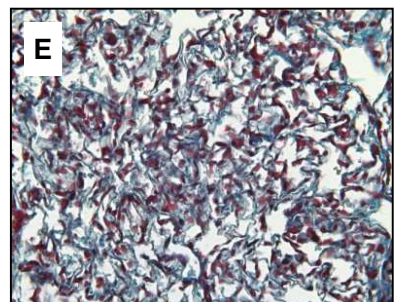

Supplement: Figure S1 — IL-1R-KO mice treated with silica particles developed limited granuloma and macrophage and neutrophil infiltration but diffuse fibrosis. Neutrophil (A) and macrophage (B) numbers in the bronchoalveolar lavage (BAL) fluid of wild-type (WT) and IL1R-knockout (IL-1R-KO) mice treated with silica (SiO2, 2.5 mg/mouse) or saline solution (NaCl) and sacrificed at day 60. Bars represent means ± SEM (n = 4–6). These results were treated statistically by a t- test. ns indicates no statistically significant difference and ** = p<0.01 indicate statistically significant difference between values measured in silica-treated WT and silica-treated KO mice. 5-µm sections of paraffin-embedded lung tissue of wild-type (WT) and IL-1R-KO mice treated with silica were stained with hematoxylin and eosin (C, 50X) or with Masson's trichrome (panels D and E, 200X). Sections are representative of 3-4 mice examined. (PDF) [file pone.0099383.s001.pdf]
